# Statins, but not proprotein convertase subtilisin‐kexin type 9 inhibitors, lower chemerin in hypercholesterolemia via low‐density lipoprotein receptor upregulation

**DOI:** 10.1002/mco2.681

**Published:** 2024-08-31

**Authors:** Lunbo Tan, Na Wang, Annet M. H. Galema‐Boers, Leonie van Vark‐van der Zee, Jeanine Roeters van Lennep, Monique T. Mulder, Xifeng Lu, A. H. Jan Danser, Koen Verdonk

**Affiliations:** ^1^ Division of Vascular Medicine and Pharmacology Department of Internal Medicine Erasmus MC Rotterdam The Netherlands; ^2^ Clinical Research Center The First Affiliated Hospital of Shantou University Medical College Shantou China

**Keywords:** chemerin, hypercholesterolemia, lipoprotein subfractions, PCSK9 inhibitors, statin

## Abstract

Hypercholesterolemia is characterized by elevated low‐density lipoprotein (LDL)‐cholesterol levels and an increased risk of cardiovascular disease. The adipokine chemerin is an additional risk factor. Here we investigated whether cholesterol‐lowering with statins or proprotein convertase subtilisin‐kexin type 9 inhibitors (PCSK9i) affects chemerin. Both statins and PCKS9i lowered plasma LDL‐cholesterol, triglycerides and total cholesterol in hypercholesterolemic patients, and increased high‐density lipoprotein (HDL)‐cholesterol. Yet, only statins additionally reduced chemerin and high‐sensitivity C‐reactive protein (hsCRP). Applying PCSK9i on top of statins did not further reduce chemerin. Around 20% of chemerin occurred in the HDL_2_/HDL_3_ fractions, while >75% was free. Statins lowered both HDL‐bound and free chemerin. Pull‐down assays revealed that chemerin binds to the HDL‐component Apolipoprotein A‐I (ApoA‐I). The statins, but not PCSK9i, diminished chemerin secretion from HepG2 cells by upregulating LDL receptor mRNA. Furthermore, chemerin inhibited HDL‐mediated cholesterol efflux via its chemerin chemokine‐like receptor 1 in differentiated macrophages. In conclusion, statins, but not PCSK9i, lower circulating chemerin by directly affecting its release from hepatocytes. Chemerin binds to ApoA‐I and inhibits HDL‐mediated cholesterol efflux. Statins prevent this by lowering HDL‐bound chemerin. Combined with their anti‐inflammatory effect evidenced by hsCRP suppression, this represents a novel cardiovascular protective function of statins that distinguishes them from PCSK9i.

## Graphical abstract

Chemerin, an adipokine and predictor of cardiovascular disease, is reduced by statins but not PCSK9 inhibitors in patients with hypercholesterolemia. This involves a direct effect of statins on chemerin release from hepatocytes, mediated via LDLR upregulation. Chemerin binds to ApoA‐I in HDL and impairs HDL‐mediated cholesterol efflux via ABCA1 and ABCG1. Chemerin‐lowering by statins will prevent this.

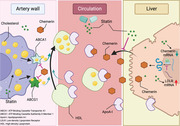

## 1 INTRODUCTION

Hypercholesterolemia is characterized by significantly elevated levels of low‐density lipoprotein cholesterol (LDL‐C) and is often caused by mutations in the low‐density lipoprotein receptor (LDLR).^1^ These mutations result in reduced or absent hepatic clearance of LDL‐C from the circulation, leading to an increased risk of atherosclerotic cardiovascular disease.^1^ Lowering cholesterol levels in hypercholesterolemia typically necessitates the use of various lipid‐lowering drugs. Traditional lipid‐lowering therapies, such as statins, inhibit the activity of HMG‐CoA reductase (HMGCR), thereby reducing cholesterol synthesis.^2^ Third‐generation statins (e.g., rosuvastatin) display greater efficacy than first‐ (pravastatin, fluvastatin) and second‐ (simvastatin, atorvastatin) generation statins.^3^ Lipophilic statins (simvastatin, fluvastatin, atorvastatin) easily diffuse into cells, while the cell entry of hydrophilic statins (pravastatin, rosuvastatin) depends on protein transporters.^3^ Statin side effects include myalgia (rarely leading to rhabdomyolysis), gastrointestinal symptoms, and liver enzyme abnormalities.^4^

A recent lipid‐lowering treatment concerns the inhibition of proprotein convertase subtilisin–kexin type 9 inhibitors (PCSK9i) with monoclonal antibodies (alirocumab, evolocumab).^2^ Since normally PCSK9 contributes to LDLR degradation, its inhibition will upregulate LDL receptor expression, thereby increasing LDL‐C clearance.^2^ Despite the high efficacy and fewer side effects of PCSK9i compared with statins, their clinical use is currently limited to patients with heterozygous familial hypercholesterolemia and patients with very high risk of atherosclerotic cardiovascular disease who require additional LDL‐C lowering.^5^, ^6^ This limitation primarily stems from the high cost and the model of administration (injection) associated with PCSK9i.^7^

The adipokine chemerin is mainly secreted by the liver, white adipose tissue, and placenta.^8^, ^9^, ^10^ Multiple lines of evidence have demonstrated that chemerin regulates adipogenesis, and lipid metabolism, and plays an important role in inflammation, mainly through its receptor chemerin chemokine‐like receptor 1 (CMKLR1).^11^, ^12^, ^13^, ^14^ Furthermore, chemerin is an independent predictor of cardiovascular events and contributes to the progression of atherosclerotic cardiovascular disease.^15^, ^16^ This involves, at least in part, chemerin‐induced upregulation of high‐sensitivity C‐reactive protein (hsCRP).^16^ Additionally, chemerin acts as a chemoattractant for CMKLR1‐expressing immune cells, facilitating their migration to sites of vascular damage,^16^ and stimulates angiogenesis, thereby exacerbating plaque growth.^16^, ^17^

Previous studies observed a positive correlation between circulating chemerin and LDL‐C, while a negative correlation was found with high‐density lipoprotein cholesterol (HDL‐C).^18^ Moreover, chemerin correlates positively with the small HDL fraction ratio, which is lowered by statins.^18^, ^19^ Interestingly, LDL apheresis in patients with hypercholesterolemia lowered chemerin in parallel with LDL.^19^, ^20^, ^21^ This suggests that chemerin is lipoprotein bound. Here, we speculate that lipid lowering in hypercholesterolemia with either statins or PCSK9i might lower chemerin, thereby additionally reducing the risk of atherosclerosis. Hence, we first compared the changes in chemerin and lipoproteins in hypercholesterolemic patients after statin and PCSK9i treatment, simultaneously focusing on the distribution of chemerin across the various lipoprotein subfractions. Second, we evaluated the effect of these drugs on chemerin release from hepatocytes, and studied whether chemerin affects cholesterol efflux in macrophages.

## 2 RESULTS

### 2.1 Clinical characteristics

The clinical characteristics of the 64 hypercholesterolemic patients who received either a statin (*n* = 25) or a PCSK9i (*n* = 39) are provided in Table 1. No significant differences between the two groups were found, except for the fact that PCSK9i users were older (*p* < 0.001), and had higher triglycerides (TG) levels (*p* < 0.05) and a higher body mass index (BMI) (*p* < 0.01). Although chemerin levels were not related to sex, LDLR mutation, or history of smoking (Table 2), they were elevated in patients who had experienced a myocardial infarction (*p* < 0.05) and in those with hypertension (*p* < 0.001). Circulating chemerin correlated positively with TG (*R* = 0.37, *p* < 0.01; Table 1), hsCRP (*R* = 0.42, *p* < 0.001), and thromobocyte levels (*R* = 0.44, *p* < 0.05), and negatively with HDL‐C and apolipoprotein A‐I (ApoA‐I) levels (*R* = −0.32 and −0.27, respectively, *p* < 0.05 for both).

**TABLE 1:** Characteristics of hypercholesterolemic patients treated with either a statin or a PCSK9i.

	PCSK9i treatment	Statin treatment	Baseline *p* value	Correlation with chemerin (no adjustment)
*N*	39	25		
Female (%)	15 (39%)	11 (48%)	0.66	
Age (years)	60 (52–68)	47 (36–55)	<0.001	0.10
BMI (kg/m^2^)	27.7 (25.7–30.0)	25.2 (23.9–27.1)	<0.01	0.08
Smoking ever (%)	25 (64%)	10 (40%)	0.12	
Mutation of LDLR (%)	18 (46%)	13 (52%)	0.65	
Myocardial infarction (%)	20 (51%)	10 (40%)	0.38	
Hypertension (%)	26 (66.7%)	12 (48%)	0.14	

							Correlation with chemerin	
	Baseline	After	Baseline	After	Baseline *p* value	Baseline	After (PCSK9i)	After (Statin)
MAP (mmHg)	100 (91–109)	NA	99 (90–107)	96 (85–101)	0.43	0.06	NA	−0.07
AST (U/L)	25 (21–29)	23 (21–32)	23 (21–31)	NA	0.72	0.02	‐0.14	NA
Triglyceride (mmol/L)	2.01 (1.40–3.12)	1.51 (0.90–2.54)	1.23 (0.93–1.85)	1.13 (0.83–1.69)	<0.05	0.37**	0.36*	0.47*
Total cholesterol (mmol/L)	6.5 (5.6–8.2)	3.5 (2.9–4.7)	7.0 (6.1–7.6)	5.0 (4.4–6.5)	0.18	0.13	0.11	0.14
HDL‐C (mmol/L)	1.17 (0.90–1.59)	1.42 (1.12–1.82)	1.27 (0.99–1.71)	1.44 (1.01–1.83)	0.11	−0.32*	−0.36*	0.10
LDL‐C (mmol/L)	4.73 (3.98–5.63)	1.52 (1.08–2.40)	5.36 (4.22–6.42)	3.05 (2.58–4.45)	0.26	−0.06	0.03	0.02
ApoA‐I (g/L)	1.33 (1.23–1.60)	NA	1.42 (1.22–1.57)	NA	0.99	−0.27*	NA	NA
ApoB (g/L)	1.47 (1.17–1.76)	0.60 (0.48–0.83)	1.56 (1.31–1.81)	0.96 (0.79–1.25)	0.35	0.08	0.06	0.24
hsCRP (ng/mL)	1434 (816–4031)	2321 (1131–4885)	1592 (742–2635)	759 (199–1443)	0.72	0.42***	0.39*	0.42*
Lp(a) (nmol/L)	30.4 (7.8–103)	NA	24.6 (9.8–38.4)	NA	0.66	0.14	NA	NA
Platelets (10^9^/L)	250 (197–281)	NA	257 (207–304)	NA	0.49	0.44*	NA	NA
Chemerin (ng/mL)	91 (76–111)	94 (71–106)	94 (78–141)	79 (56–99)	0.31			

Values are presented as either *N* number (and percentage of total) or median and interquartile range (25% percentile–75% percentile).

Abbreviations: ApoA‐I, apolipoprotein A‐I; ApoB, apolipoprotein B; AST, aspartate transaminase; BMI, body mass index; HDL‐C, high‐density lipoprotein cholesterol; hsCRP: high‐sensitivity C‐reactive protein; LDL‐C, low‐density lipoprotein cholesterol; Lp(a), lipoprotein(a); MAP, mean arterial pressure; NA, no data. The correlation with chemerin was analyzed by the Spearman's method and adjusted for sex, age and BMI.

*p < 0.05, **p < 0.01, ***p < 0.001.

**TABLE 2:** Chemerin levels in hypercholesterolemic patients according to sex, LDLR mutation, history of smoking, and the occurence of a myocardial infarction or hypertension.

	Chemerin level (ng/mL)		
Parameter	No	Yes	*p*‐Value
Female sex	84 (66–111)	92 (79–135)	0.12
Mutation of LDLR	93 (75–124)	84 (72–104)	0.48
Smoking ever	84 (66–113)	93 (78–127)	0.16
Myocardial infarction	84 (67–115)	99 (82–147)	<0.05
Hypertension	79 (59–96)	99 (80–135)	<0.001

Values are median and interquartile range (25% percentile–75% percentile).

LDLR, low‐density lipoprotein cholesterol receptor. Statistical comparison was by Mann–Whitney *U*‐test. NS, nonsignificant. No and yes refer to the absence/presence of the parameter in the left part of the table.

Table S1 provides the clinical characteristics of 23 hypercholesterolemic patients that received a PCSK9i (alirocumab *n* = 12, evolocumab, *n* = 11) on top of a statin (fluvastatin *n* = 5, simvastatin *n* = 6, atorvastatin *n* = 6, and rosuvastatin *n* = 6). Their age and BMI were comparable to those receiving PCSK9i only.

### 2.2 Effects of statins and PCSK9i on lipid levels and inflammatory markers

**FIGURE 1:**
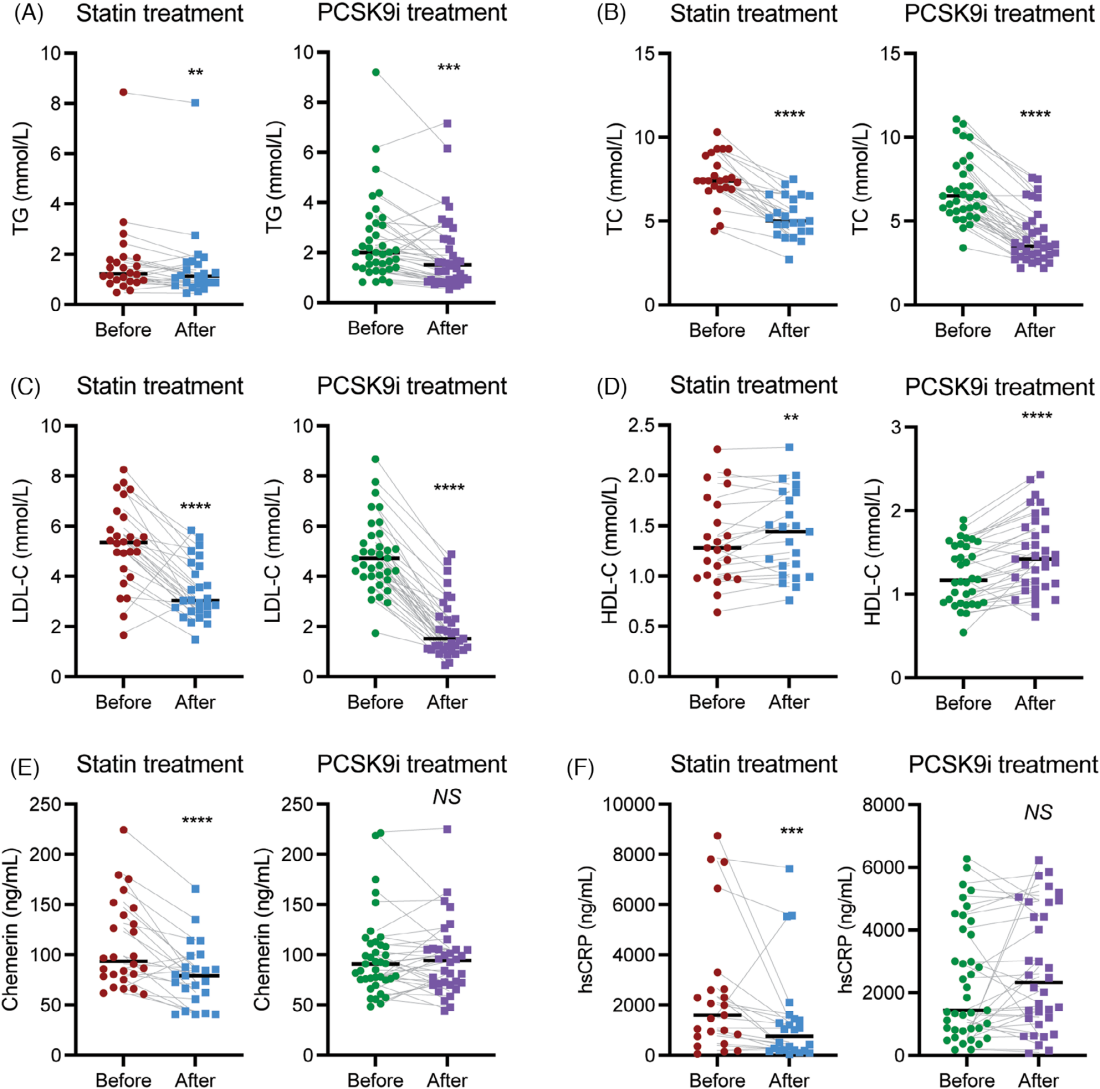
Lipids and inflammatory markers in hypercholesterolemic patients before and after statin or proprotein convertase subtilisin‐kexin type 9 inhibitors (PCSK9i) treatment. Panels A–F display the individual levels and median values of plasma triglycerides (TG), total cholesterol (TC), low‐density lipoprotein cholesterol (LDL‐C), high‐density lipoprotein cholesterol (HDL‐C), chemerin, and high‐sensitivity C‐reactive protein (hsCRP) in 39 PCSK9i users and 25 statin users before and after treatment. NS, not significant, **p < 0.01, ***p < 0.001, and ****p < 0.0001 versus before.

Statins and PCSK9i administration resulted in the expected effects on lipids (Figure 1A–D), that is, decreases in TG, total cholesterol (TC), and LDL‐C, and an increase in HDL‐C (*p* < 0.01 for all). Yet, only statin treatment reduced chemerin (*p* < 0.0001; Figure 1E) and hsCRP (*p* < 0.001; Figure 1F). Additionally, the effect of PCSK9i on TG (*p* < 0.05), TC (*p* < 0.001), LDL‐C (*p* < 0.0001), and HDL‐C (*p* < 0.01) was larger than that of statins. The reductions in LDL‐C and chemerin were unrelated to sampling time (*R* = 0.14, *p* = 0.24; *R* = −0.16, *p* = 0.17, respectively). Furthermore, all above described correlations with circulating chemerin remained unaltered after treatment (Table 1), except the negative correlation between chemerin and HDL‐C which disappeared after statin treatment. Table S2 shows that the statin effect on chemerin is seen with fluvastatin, simvastatin, and atorvastatin, but not rosuvastatin.

In patients that received PCSK9i treatment on top of a statin, further decreases in TG (*p* < 0.05), TC (*p* < 0.0001), apolipoApoB (*p* < 0.0001), and LDL‐C (*p* < 0.0001) were observed, while HDL‐C (*p* < 0.01) increased (Table S1 and Figure S1). Yet, there was no change in either chemerin or hsCRP. Dual treatment did not affect the positive correlation between chemerin and TG, while the correlation between chemerin and HDL‐C now became positive (*R* = 0.45, *p* < 0.05).

In summary, both lipid‐lowering therapies exerted their expected effects on lipids, but only statins additionally lowered chemerin and hsCRP.

### 2.3 Chemerin distribution across lipoprotein subfractions

**FIGURE 2:**
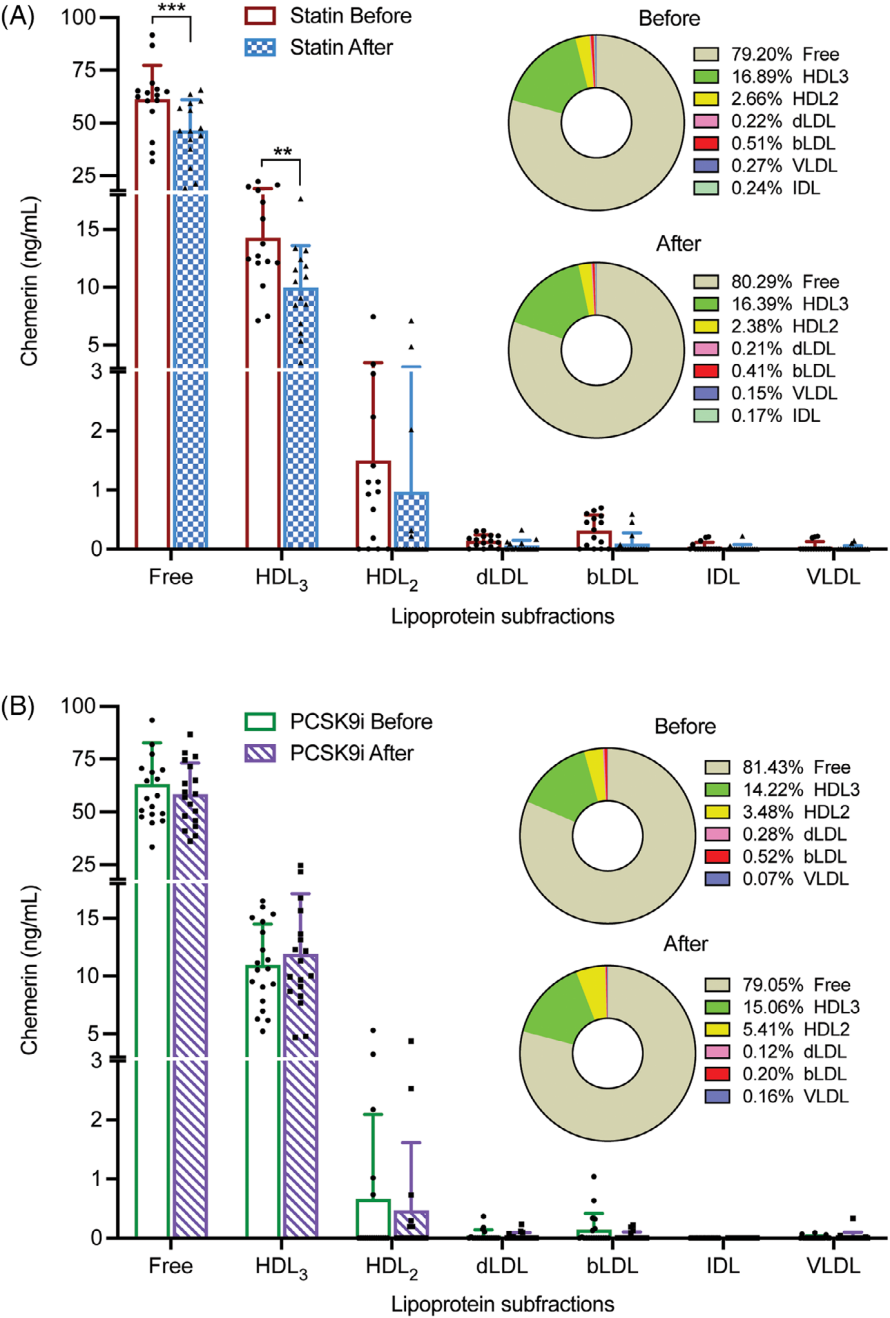
Free versus lipoprotein fraction‐bound chemerin levels before and after statin (panel A) or PCSK9i (panel B) treatment. Lipoprotein subfractions were isolated from plasma of hypercholesterolemic patients using density‐gradient ultracentrifugation. The circles on the right display its distribution across the various fractions. Data are mean ± SD of 15 statin users and 19 PCSK9i users. **p < 0.01, ***p < 0.001 versus before.

At baseline, in both treatment groups more than 75% of chemerin was in the free form, that is, unbound to lipoproteins (Figure 2). Among the lipoproteins fractions, chemerin occurred predominantly in the HDL_3_ and HDL_2_ fractions. Treatment did not alter this distribution, and reductions in absolute amounts occurred only in the free (*p* < 0.001) and HDL_3_ (*p* < 0.01) fractions after statin treatment, while a similar tendency was observed for the HDL_2_ fraction (*p* = NS). Taken together, these data indicate that the statin‐induced reduction in chemerin is a general phenomenon, that is, it is not limited to a specific fraction.

### 2.4 Identification of chemerin–ApoA‐I interaction in hypercholesterolemia plasma

**FIGURE 3:**
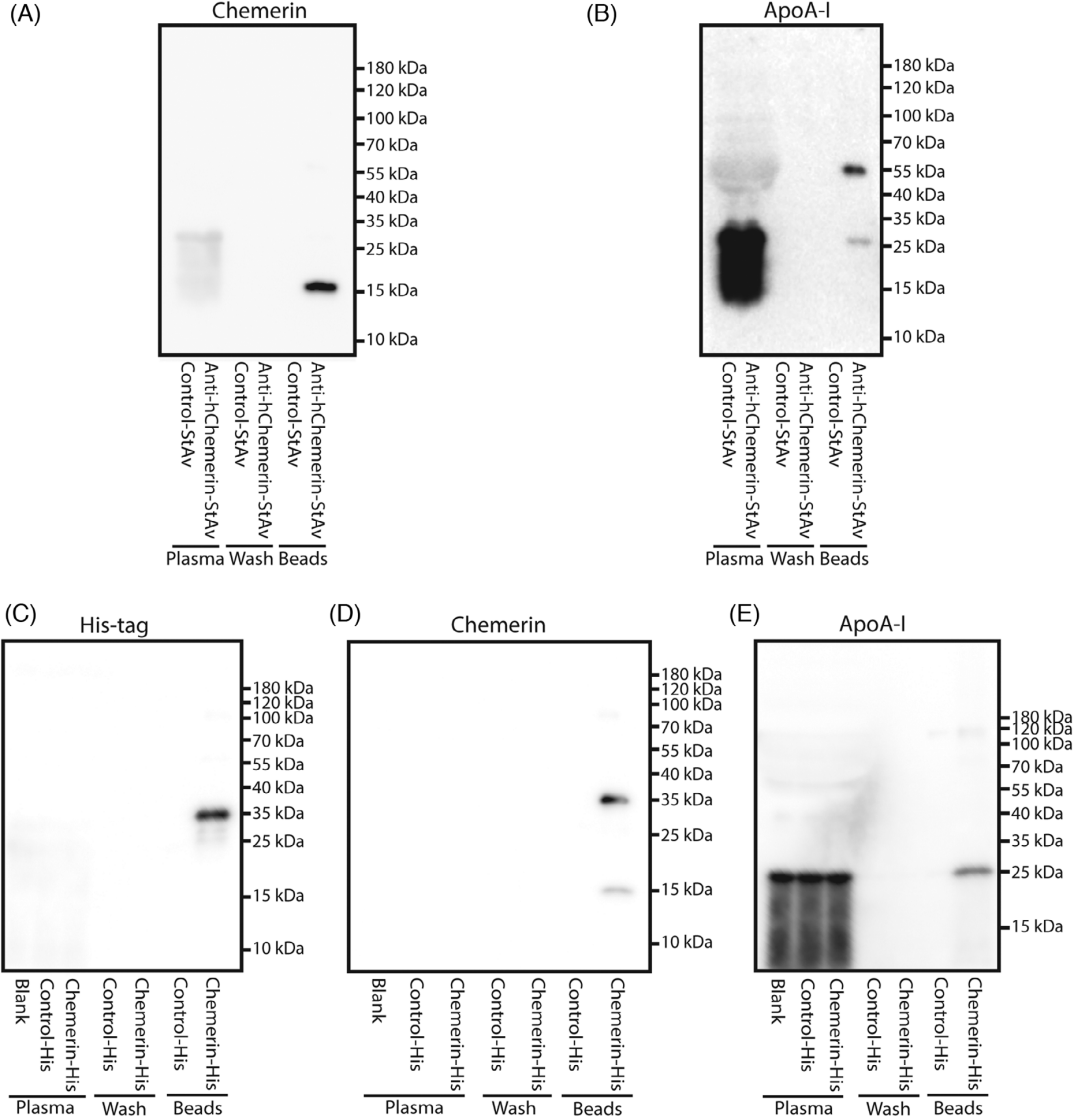
Pull‐down assay supports chemerin‐apolipoprotein A‐I (ApoA‐I) interaction in plasma of hypercholesterolemic patients. Plasma was incubated with streptavidin‐labeled anti‐human chemerin, and panels A and B show the specific binding of endogenous chemerin and ApoA‐I in the elution fluid of the streptavidin beads. When incubating plasma with exogenous chemerin with a His‐tag, the elution fluid of the His‐tag beads subsequently contained the His tag, chemerin and ApoA‐I (panels C–E).

The chemerin occurrence in the smaller HDL fractions might imply that chemerin interacts with ApoA‐I.^18^, ^19^ To investigate such interaction, streptavidin‐labeled anti‐human chemerin was added to a hypercholesterolemic patient plasma pool. Chemerin could be detected in the elution fluid of the streptavidin beads, and this was also true for ApoA‐I (Figure 3A,B). Moreover, when supplementing the plasma pool with human chemerin‐His protein, His tag, chemerin and ApoA‐I were subsequently detected in the elution fluid of His‐tag beads (Figure 3C–E). In summary, these data support physical chemerin–ApoA‐I interaction in hypercholesterolemic plasma.

### 2.5 Statins and PCSK9i counteract the inhibitory effect of chemerin on the cholesterol efflux ability of HDL

**FIGURE 4:**
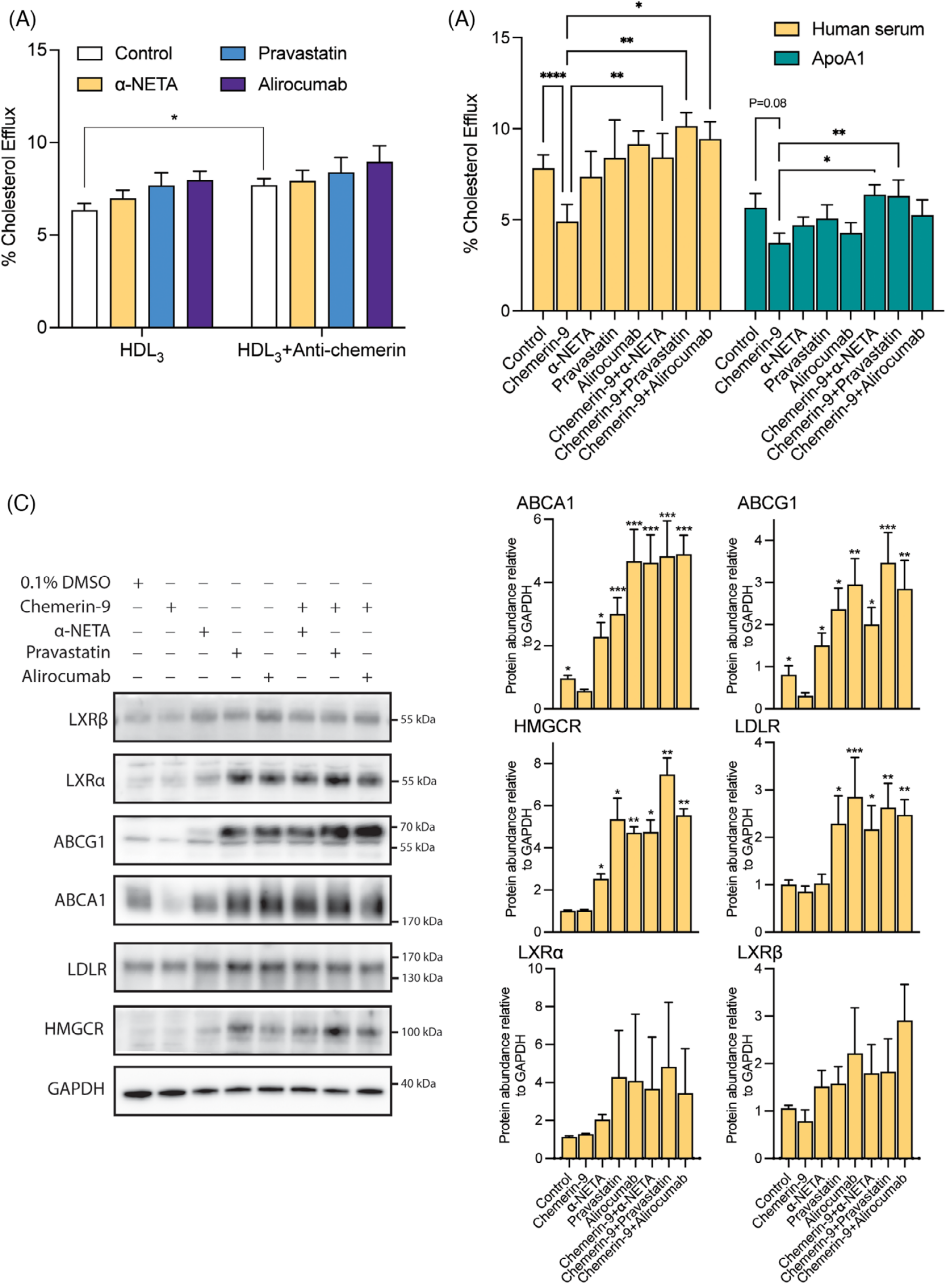
The impact of chemerin on cholesterol efflux in macrophages. In panel A, the cholesterol efflux assay was performed on differentiated THP‐1 cells, using HDL_3_ isolated via ultracentrifugation from plasma of hypercholesterolemic patients as the cholesterol acceptor. Experiments occurred in the presence or absence of 10 ng/mL anti‐human chemerin antibody, with or without 3 µmol/L α‐NETA, 20 µmol/L pravastatin, or 30 µg/mL alirocumab. Data are mean ± SEM, *n* = 3, *p < 0.05. Efflux was calculated as medium fluorescence/(medium fluorescence + cell lysate fluorescence). In panel B, 100 nmol/L chemerin‐9, the active isoform of chemerin, was used to assess the effect of chemerin on cholesterol efflux from differentiated THP‐1 cells, in the presence or absence 3 µmol/L α‐NETA, 20 µmol/L pravastatin, or 30 µg/mL alirocumab, using as cholesterol acceptors either 2 µL human serum or 20 µg/mL recombinant human ApoA‐I. Data are mean ± SEM, *n* ≥ 5. *p < 0.05, **p < 0.01, ***p < 0.001, ****p < 0.0001 versus chemerin‐9 alone. Panel C show the levels of cholesterol efflux‐related proteins after the chemerin‐9 incubations for 6 h. LXRα and LXRβ, liver X receptors α and β; ABCA1, ATP Binding Cassette subfamily A member 1; ABCG1, ATP Binding Cassette subfamily G member 1; LDLR, low‐density lipoprotein receptor; HMGCR, HMG‐CoA reductase. Data are mean ± SEM, *n* = 5. *p < 0.05, **p < 0.01, ***p < 0.001, ****p < 0.0001 versus chemerin‐9 alone.

Chemerin–ApoA‐I interaction could be involved in the HDL‐induced cholesterol efflux response. We tested this hypothesis in differentiated THP‐1 cells. After 24 h of incubation, chemerin was below detection limit in the medium of differentiated THP‐1 cells, but easily detectable when the HDL_3_ fraction had been added to the medium (Figure S2A). HDL_3_ fraction addition decreased the key proteins ATP Binding Cassette subfamily A member 1 (ABCA1) and ATP Binding Cassette subfamily G member 1 (ABCG1) (Figure S2B), indicating inhibition of cholesterol transport. Moreover, adding the HDL_3_ fraction increased CMKLR1 but not chemerin receptor C‐C motif chemokine receptor‐like 2 (CCRL2; Figure S2B). The anti‐human chemerin antibody enhanced cholesterol efflux during incubation with the HDL_3_ fraction (Figure 4A), and this was not altered by α‐NETA, pravastatin, or alirocumab. Alirocumab even increased CMKLR1 under these conditions (Figure S2B). To investigate the effect of chemerin on cholesterol transport in differentiated THP‐1 cells, chemerin‐9, the active isoform of chemerin, was used. Chemerin‐9 inhibited cholesterol efflux in the presence of both human serum and human ApoA‐I (serving as the cholesterol acceptor) when compared with vehicle (Figure 4B). The CMKLR1 antagonist α‐NETA, pravastatin, atorvastatin, rosuvastatin, alirocumab, and evolocumab prevented this effect in the presence of serum, while only α‐NETA and pravastatin prevented it in the presence of ApoA‐I (Figures 4B and S3). Western blot analysis of cell lysates (Figure 4C) revealed that all antagonists, either with or without chemerin‐9, upregulated HMGCR, ABCA1, and ABCG1, while a similar tendency was shown for liver X receptors α and β (LXRα and LXRβ). In addition, alirocumab and pravastatin, both alone and in the presence of chemerin‐9, upregulated LDLR. Taken together, these data indicate that chemerin inhibits cholesterol efflux via CMKLR1, and that both statins and PCSK9i prevent this effect, potentially by interfering with key proteins involved in cholesterol transport (ABCA1 and ABCG1).

### 2.6 Statins, not PCSK9i, suppresses chemerin release from hepatic cells

**FIGURE 5:**
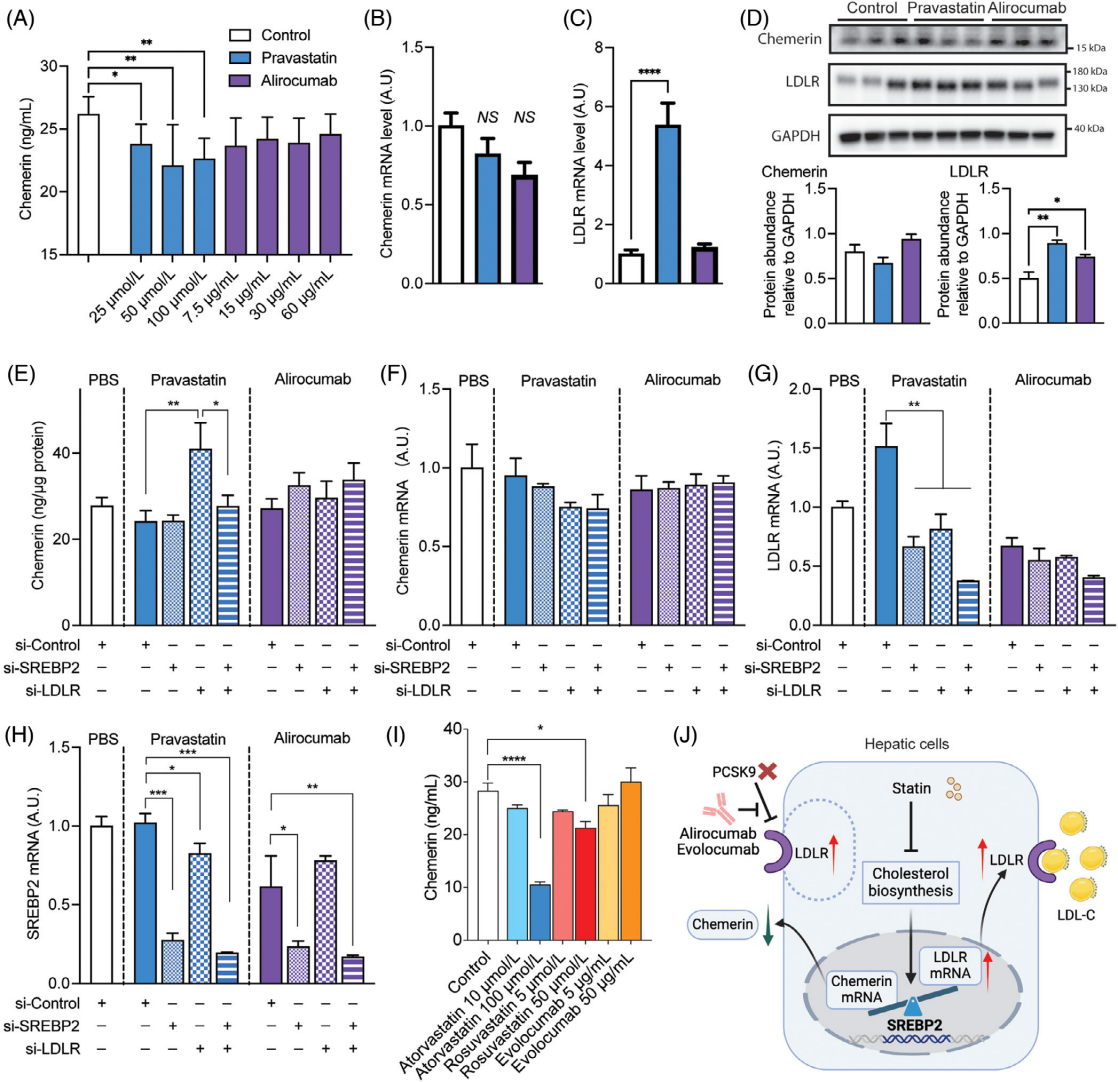
The impact of statins and PCSK9i on the release of chemerin from HepG2 cells. Panel A, chemerin levels in the medium of HepG2 cells cultured for 48 h, in the absence (control) or presence of pravastatin or alirocumab. Data are mean ± SEM of *n* ≥ 5. Panels B–D, chemerin and LDLR mRNA expression and protein abundance in HepG2 cells cultured for 48 h, in the absence (control) or presence of 50 µmol/L pravastatin or 30 µg/mL alirocumab. Data are mean ± SEM of *n* ≥ 5. NS, not significant, *p < 0.05, **p < 0.01, ****p < 0.0001 versus control. Panel E, chemerin in the medium relative to total protein of HepG2 cells cultured for 48 h under the following conditions: in the absence (control) or presence of pravastatin or alirocumab, and with or without the application of control, SREBP2, or LDLR siRNA (si). Data are mean ± SEM of *n* = 3. *p < 0.05, **p < 0.01 versus si‐Control. Panels F–H, chemerin, LDLR, and SREBP2 mRNA expression in HepG2 cells cultured for 48 h under the following conditions: in the absence (control) or presence of pravastatin or alirocumab, and with or without the application of control, SREBP2, or LDLR siRNA (si). Panel I, chemerin levels in the medium of HepG2 cells cultured for 48 h, in the absence (control) or presence of atorvastatin, evolocumab, or rosuvastatin. Data are mean ± SEM of *n* ≥ 3. Panel J, unifying scheme, see text for explanation. SREBP2, sterol‐regulatory‐element‐binding protein‐2; LDLR, low‐density lipoprotein receptor.

Given the hepatic origin of circulating chemerin,^22^ we used HepG2 cells to verify whether statins or PCSK9i directly affect chemerin synthesis. As expected, HepG2 cells release chemerin in a time‐dependent manner (Figure S4A). Chemerin could not be detected in fetal bovine serum (FBS)‐containing medium in the absence of cells (Figure S4A), excluding that its release represented chemerin in FBS. Pravastatin, but not alirocumab, concentration‐dependently suppressed the release of chemerin into the medium (Figure 5A), and the degree of suppression (around 15−20%) mimicked the in vivo suppression of circulating chemerin by statins (Figure 1E). Neither drug affected cellular chemerin (Figure 5D), and although both drugs tended to decrease chemerin gene expression (Figure 5B), this was not significant. Only pravastatin upregulated LDLR gene expression (by 436%, *p* < 0.0001; Figure 5C), while both pravastatin (*p* < 0.01) and alirocumab (*p* < 0.05) at most modestly upregulated LDLR at the protein level (Figure 5D). This implies that the increase in LDLR gene expression after pravastatin did not result in a parallel increase in LDLR protein levels. No change in the unfolded protein response‐related proteins glucose‐regulated protein 78, CCAAT‐enhancer‐binding protein homologous protein, protein kinase R‐like endoplasmic reticulum kinase, X‐box binding protein 1, activating transcription factor 6, and eukaryotic translation initiation factor 2 subunit 1 was observed (Figure S4B,C). This argues against an alteration in the accumulation of unfolded or misfolded proteins as an explanation of the effect of pravastatin.

Since the transcription factor sterol‐regulatory‐element‐binding protein 2 (SREBP2) is responsible for both LDLR and chemerin transcription, one further possibility is that upregulating LDLR transcription after statin exposure, diminishes concomitant chemerin transcription. In agreement with this concept, knockdown of LDLR during incubation with pravastatin even increased chemerin release, while additional SREBP2 knockdown prevented this effect (Figure 5E). LDLR knockdown, SREBP2 knockdown, or both during pravastatin or alirocumab exposure did not affect chemerin mRNA levels (Figure 5F), although they did reduce LDLR mRNA levels during pravastatin exposure (Figure 5G). As expected, SREBP2 knockdown diminished the SREBP2 mRNA levels during both pravastatin and alirocumab exposure (Figure 5H). Cell lysate data revealed that pravastatin during LDLR knockdown upregulated SREBP2 and that additional SREBP2 knockdown prevented this (Figure S4D). No such observations were made during incubation with alirocumab, although LDLR knockdown during alirocumab incubation did downregulate the LDLR protein level to the same degree as during pravastatin incubation.

Finally, in a concentration‐dependent manner, both atorvastatin and rosuvastatin, but not evolocumab, were also capable of decreasing chemerin release into the medium (Figure 5I).

In summary, these data imply that statins, but not PCSK9i, diminish chemerin secretion from HepG2 cells, and that this involves LDLR upregulation.

## 3 DISCUSSION

While the role of statins and PCSK9i in regulating blood lipid levels, including the reduction of TC and LDL‐C, and the augmentation of HDL‐C, is well established, our current research illuminates intriguing additional dynamics. Specifically, statins might confer an extra advantage over PCSK9i by attenuating chemerin and hsCRP levels. The chemerin suppression by statins involved a reduction in both free chemerin and its occurrence in the HDL fraction. The latter is reinforced by the evidence demonstrating a physical interaction between chemerin and ApoA‐I. We also found that chemerin exerts a detrimental effect on cholesterol efflux capacity in macrophages. This inhibition could be counteracted by both statins and PCSK9i through the upregulation of ABCA1 and ABCG1. A critical point to underscore is that statins, unlike PCSK9i, inhibit the release of chemerin from hepatic cells in vitro, reaching a degree of suppression (15–20%) that is similar to the suppression in circulating chemerin observed after statin treatment of hypercholesterolemic patients.

Chemerin was initially discovered as a chemokine and was found to play a significant role in immune cells. However, subsequent research confirmed its impact on lipid metabolism, establishing it as an adipokine.^13^, ^23^ The exact cause of elevated chemerin levels remains uncertain, as it could be induced by changes in both lipids and inflammatory conditions. Clinical investigations have revealed correlations between chemerin and circulating lipid levels, as well as inflammatory protein levels. For instance, in obese and healthy individuals, chemerin associates positively with LDL‐C, BMI, TG, TC, interleukin‐6, tumor necrosis factor‐α, and CRP, while demonstrating a negative association with HDL‐C.^24^, ^25^, ^26^ However, in patients with rheumatoid arthritis and non‐small cell lung cancer, the positive correlation between chemerin and LDL‐C, BMI, TG, and TC either disappeared, or turned into a negative correlation.^24^, ^27^, ^28^ In the present study in hypercholesterolemic patients, we also observed positive correlations between plasma chemerin and TG, and hsCRP, and a negative correlation with HDL‐C. The negative association with HDL‐C disappeared after treatment with statins and remained after treatment with PCSK9i. Clearly, the effect of chemerin may vary among different patient populations and may be affected by distinct physiological events and pharmacological treatment.

Hypercholesterolemic patients face a notable risk of developing atherosclerotic cardiovascular disease resulting from a combination of lipid abnormalities and inflammation.^29^ Chemerin is known as an independent risk factor for cardiovascular events, as it is implicated in various aspects of atherosclerosis.^15^ It positively affects the formation of atherosclerotic vascular lesions while also contributing to endothelial dysfunction and arterial stiffness.^16^, ^30^ Additionally, chemerin is involved in the orchestration of the chronic inflammatory process and the development of atherosclerosis.^16^, ^30^ It does so by modulating endothelial cell adhesion molecules, enabling stronger adhesion between macrophages, lymphocytes, and endothelial cells, thus advocating for M1 macrophage polarization.^16^ The negative association between chemerin and HDL‐C, and the positive association between chemerin and hsCRP and thrombocytes supports its role in the pathogenesis of atherosclerosis in hypercholesterolemic patients. Additionally, elevated chemerin levels were observed in hypercholesterolemic patients who had experienced cardiovascular events, specifically myocardial infarction and hypertension, highlighting its potential as a prognostic marker with clinical significance.

In clinical practice, the primary treatment objective for hypercholesterolemia is to achieve cholesterol reduction and thereby minimize the risk of cardiovascular events.^5^ Among available therapeutic options, statins have emerged as the most efficacious agents for lowering LDL‐C levels across populations at risk for cardiovascular disease.^31^ Statin therapy, despite its proven benefits, often sees discontinuation due to a range of factors. These include adverse effects, lack of sufficient follow‐up, and problems associated with treatment adherence.^6^ As an alternative, PCSK9i, delivered as PCSK9‐inactivating antibodies, present an appealing option.^6^ They are associated with fewer side effects and have shown substantial capacity to reduce LDL‐C levels.^2^, ^6^ As a result, they are receiving increasing attention and uptake in clinical practice.^6^ In the present study, PCSK9i reduced TG, LDL‐C, and TC levels more strongly than statins. Yet, statins demonstrated a more pronounced anti‐inflammatory effect, as evidenced by a reduction in hsCRP levels. These findings concur with previous research conducted on PCSK9i (cococizumab, evolocumab, or alirocumab) and statins (atorvastatin, rosuvastatin, or simvastatin).^2^ Furthermore, it is noteworthy that statin treatment led to a decrease in chemerin levels, whereas no such effect was observed with PCSK9i treatment. This discrepancy may potentially indicate a divergence in the cardiovascular protective mechanisms employed by these two therapeutic approaches.

The central action of statins, but not PCSK9 inhibitors, in reducing chemerin levels can largely be ascribed to their capacity to inhibit the release of chemerin from liver cells, the established primary source of circulating chemerin.^22^ Our experiments revealed that all statins investigated in this study (pravastatin, atorvastatin, and rosuvastatin) but not the PCSK9i alirocumab and evolocumab lowered the release of chemerin from liver cells. The cellular content of chemerin was very low (suggestive for immediate release and no storage), and unaffected by either drug, although both drugs tended to lower chemerin gene expression. However, gene expression changes do not necessarily underlie alterations in secretion.^32^, ^33^ Interestingly, pravastatin highly significantly upregulated LDLR gene expression (>fivefold), while only modestly upregulating the LDLR protein level (1.5‐fold). Alirocumab also upregulated the LDLR protein level. The latter is expected since PCSK9i slows down LDLR degradation. The rise in LDLR gene expression after pravastatin most likely reflects an indirect effect, due to cholesterol lowering.^2^ Here it is important to mention that the transcription factor SREBP2 is responsible for both LDLR and chemerin transcription.^14^, ^34^ Thus, if significant LDLR upregulation occurs (like after statin treatment, but not after alirocumab treatment), less SREBP2 will be available for chemerin transcription, so that the latter drops. Our studies in HepG2 cells support this concept, and even show that in the absence of the LDLR (after its knockdown by siRNA) pravastatin, by upregulating SREBP2, increased chemerin release. Despite previous reports linking statins with effects on unfolded protein responses,^35^, ^36^ our study found no such association. Therefore, at present the most likely explanation of pravastatin's suppression of chemerin release concerns its induction of LDLR gene expression in liver cells, effectively reducing the availability of the transcription factor SREBP2 (Figure 5G). Nevertheless, knockdown of SREBP2 alone did not affect chemerin expression, implying that mechanisms beyond SREBP2 availability should also be considered.

Apheresis lowers LDL‐C and chemerin in parallel and also reduces small HDL.^21^ The latter observation has also been made for pravastatin.^20^ This finding, combined with correlations observed between chemerin and small LDL and small HDL,^18^ prompted us to verify the interaction between chemerin and lipoproteins. To this end, we quantified chemerin in ultracentrifugation (UC)‐separated plasma samples of hypercholesterolemic patients. We found that around 20% of plasma chemerin was present in the HDL_2_ and HDL_3_ fractions, while the majority (>75%) was nonlipoprotein bound. Given the fact that ApoA‐I is the major apolipoprotein in the HDL fraction, we next used a pull‐down approach to confirm that chemerin physically binds to HDL. Finally, we showed that chemerin inhibits the HDL‐induced cholesterol efflux via CMKLR1. Both pravastatin and alirocimuab prevented this effect, potentially by upregulatingkey proteins involved in cholesterol transport (ABCA1 and ABCG1). However, in vivo, only pravastatin lowered the chemerin content of the HDL fractions. Taken together, these data imply that small HDL in hypercholesterolemic patients can load chemerin, thereby altering its effect on cholesterol efflux. Statins might prevent this phenomenon, at least in part, by reducing the chemerin content of small HDL. This mechanism could explain why chemerin acts atherogenic.^37^ Future studies should address whether the reduced occurrence of chemerin in the HDL_2/3_ fraction after statin treatment simply is the result of diminished chemerin synthesis in the liver, or additionally involves a direct effect on chemerin–ApoA‐I interaction.

This study has several limitations. First, our human data are obtained from a relatively small number of patients and involve multiple different statins and PCKS9i. Larger cohorts might help to verify whether the observed effects truly are class effects. Second, although we did not find an effect of LDLR mutations on chemerin levels in hypercholesterolemic patients, future studies should sort out whether upregulated LDLR gene expression after statin treatment truly lowers chemerin protein level, for example, via competition for SREBP2, and to what degree this is affected by specific LDLR mutations. Third, it is crucial to investigate what facilitates chemerin–HDL interaction. Here, inflammatory factors might play a role, as well as statins themselves. Finally, we need to know the long‐term consequence of chemerin‐lowering, for example, on blood pressure and the occurrence of a myocardial infarction. Such knowledge would offer valuable insights for the management of patients at high risk of cardiovascular events. In conclusion, we found that both statins and PCSK9 inhibitor are effective in modulating blood lipid levels in hypercholesterolemic patients, with statins offering additional benefits by inhibiting chemerin and hsCRP levels and influencing HDL‐chemerin interactions. This may represent a novel cardiovascular protective function of statins.

## 4 METHODS

### 4.1 Study population

Patients (*n* = 87) diagnosed with hypercholesterolemia and attending the outpatient clinic at Erasmus Medical Center in Rotterdam, the Netherlands, between October 2011 and October 2019 were included in this study. A total of 64 of patients received either a statin, or, in case of statin intolerance (defined as statin‐associated myalgia for at least three different statins, in accordance with the criteria described by the European Atherosclerosis Society/European Society of Cardiology consensus),^38^ a PCSK9i. A total of 23 patients received statin treatment combined with a PCSK9i. All hypercholesterolemic patients receiving a PCSK9i met the Dutch criteria for treatment with such a drug, that is, having an LDL‐C level > 2.5 mmol/L without cardiovascular disease events or an LDL‐C level > 1.8 mmol/L with a history of cardiovascular disease events.^38^ Figure S5 provides the flowchart for patient inclusion from our existing open cohort study.^39^ Hypercholesterolemia diagnosis was based on a documented LDL receptor mutation or clinical hypercholesterolemia criteria (Dutch Lipid Clinic Network score ≥6).^40^ The category “normal blood cholesterol” was defined as having a TC < 5 mmol/L and LDL‐C < 3 mmol/L, and not being on blood lipid‐lowering treatment.^38^, ^40^ In agreement with standard clinical dose recommendations, statin treatment involved simvastatin, fluvastatin, rosuvastatin, or atorvastatin (40, 80, 20, and 20 mg per day, respectively), while PCSK9i treatment involved alirocumab or evolocumab (140 and 150 mg every 2 weeks, respectively).^2^ A follow‐up visit occurred between 30 and 250 days after initiation of the therapy. Venous blood samples were collected in EDTA tubes after an overnight fast both at the start of therapy and during follow‐up. Blood was centrifuged at 2500×*g*, and the resulting plasma was stored at −80°C until analysis. Anthropometric data were obtained from medical records. Participants’ medical history, family history, and lifestyle information were assessed via a questionnaire. Hypertension was defined as systolic blood pressure > 140 mmHg and/or diastolic blood pressure > 90 mmHg, or the use of antihypertensive therapy. Mean arterial pressure was calculated using the formula (2 × diastolic pressure + systolic pressure)/3. Participants were categorized as smokers or never smokers based on their self‐reported smoking status.

### 4.2 Lipoprotein subfraction isolation

Plasma lipoprotein subfractions were separated using the density‐gradient UC method described previously.^41^ The density gradient was prepared by adding KBr (0.35 g/mL) to plasma, resulting in a density of 1.26 g/mL, of which 1 mL was placed in an ultracentrifuge tube. On top of this layer, KBr solutions with densities of 1.21, 1.10, 1.063, 1.04, and 1.02 g/mL, each comprising 1.9 mL, were sequentially added, followed by 1 mL of water. The samples were then ultracentrifuged at 221,633x*g* for 18 h at 4°C using a SW41 rotor in an L‐40 Beckman ultracentrifuge (Beckman Instruments, USA). The resulting density gradient was fractionated, starting from the bottom of the tube, into 250 µL fractions. The fractions with densities of 1.26–1.21 g/mL were designated as free protein without lipoprotein binding level (Free). Fractions with densities of 1.125−1.21 and 1.062−1.125 g/mL were identified as HDL_3_ and HDL_2_, respectively. Additionally, fractions with densities ranging from 1.038−1.063 and 1.019−1.038 g/mL were characterized as small‐dense LDL (dLDL) and buoyant LDL (bLDL), respectively. Fractions with a density ≤ 0.971 g/mL represented VLDL.^42^

### 4.3 Cell culture

HepG2 and THP‐1 cells were obtained from the American Type Culture Collection (ATCC, Manassas, VA, USA). HepG2 cells were cultured in complete medium with Eagle's Minimum Essential Medium (ATCC) and 10% FBS (GE Healthcare, Eindhoven, the Netherlands) in T‐75 flasks (Costar, Corning, NY, USA) at 37°C with 5% CO_2_. The medium was refreshed every 2−3 days and the cells were subcultured at 80−85% confluence. THP‐1 cells were maintained in complete medium composed of RPMI‐1640 (ATCC) supplemented with 10% FBS and 0.05 mmol/L 2‐mercaptoethanol (Gibco, Grand Island, NY, USA) in T‐75 flasks (Costar) at 37°C with 5% CO_2_. The use of the initial thawed vial was limited to 10 passages to control for genetic drift in the experiments.

#### 4.3.1 Chemerin production in liver cells

HepG2 cells were cultured in 24‐well plates (Costar) with complete medium. Medium was collected at 24, 48, and 72 h for the measurement of chemerin. To investigate the effect of pravastatin (Sigma–Aldrich, Saint Louis, MO, USA), atorvastatin (MCE, Monmouth Junction, NJ, USA), rosuvastatin (MCE), alirocumab (PRALUENT, Tarrytown, NY, USA), or evolocumab (MCE) on chemerin production, cells were cultured in the presence of 50 µmol/L pravastatin, 10 and 100 µmol/L atorvastatin, 5 and 50 µmol rosuvastatin, 30 µg/mL alirocumab, or 5 and 50 µg/mL evolocumab for 48 h in 12‐well plates, after which the medium was collected. Cells were lysed with either TRIzol (Invitrogen, Breda, The Netherlands) or RIPA lysis buffer (Thermo Fisher Scientific, Waltham, MA, USA) for the quantification of chemerin mRNA and protein expression, by real‐time PCR and western blot, respectively. To study the role of SREBP2 and LDLR, HepG2 cells in 24‐well plates for 48 h were transfected with 20 nmol/L SREBP2 and LDLR siRNA (both from Thermo Fisher Scientific; siRNA ID's 107467 and 106132, respectively) using RNAiMAX according to the manufacturer's instructions,. A scrambled siRNA sequence (Thermo Fisher Scientific; AM4611) was used as a nonspecific control, also at a final concentration of 20 nmol/L.

#### 4.3.2 Cholesterol efflux assay

THP‐1 monocytes were seeded at 2.5 × 10^5^ cells/mL in either 96‐well plates (Costar) for the cholesterol efflux assay or 12‐well plates (Costar) for western blotting. The cells were cultured in complete medium supplemented with 50 ng/mL of phorbol 12‐myristate 13‐acetate (Thermo Fisher Scientific) for 72 h to induce differentiation into macrophages. Differentiated cells were subjected to a cholesterol efflux assay using a commercial kit (Sigma–Aldrich; MAK192‐1KT) according to the manufacturer's instructions. Briefly, the cells were labeled with a labeling mix for 1 h and then equilibrated with an equilibration mix overnight (16 h). The equilibration mix was aspirated, and the cells were washed with serum‐free RPMI medium. Cholesterol acceptors were 2 µL human serum (Sigma–Aldrich; H4522, from human male AB plasma, USA origin, sterile‐filtered) with 27.5 ng/mL of chemerin, 20 µg/mL recombinant human apolipoprotein A1 (ApoA‐I; Sigma–Aldrich), or 10 µL HDL_3_ isolated via UC from a plasma pool (see below), generated by mixing 300‐µL plasma samples obtained at baseline from 10 individual hypercholesterolemic patients (five statin users and five PCSK9i users, aged 50−60 years). The acceptors were added to the cells in the presence of PBS (control), 100 nmol/L chemerin‐9 (MCE), or 10 ng/mL anti‐human chemerin antibody (Thermo Fisher Scientific), with or without 3 µmol/L of the CMKLR1 antagonist α‐NETA (MCE), 20 µmol/L pravastatin, 100 µmol/L atorvastatin, 50 µmol/L rosuvastatin, 30 µg/mL alirocumab, or 50 µg/mL evolocumab. Next, the cells were incubated for 6 h at 37°C with 5% CO_2_. At the end of the incubation, the supernatant of each well was transferred to a new 96‐well plate and its fluorescence was measured (Fm, λEx = 485/λEm = 523 nm). The cell monolayer was solubilized with cell lysis buffer and its fluorescence was also measured (Fc, λEx = 485/λEm = 523 nm). The cholesterol efflux rate *C* was calculated as follows: *C* = 100% × Fm/(Fm + Fc). All samples were run in triplicates. Data were corrected for the efflux rate determined in the absence of cholesterol acceptor. For western blotting, the differentiated cells were cultured in RPMI‐1640 supplemented with 10% human serum (Sigma–Aldrich) and 0.05 mmol/L 2‐mercaptoethanol, and exposed to the same treatments mentioned above for 6 h, after which the cell lysates were collected.

### 4.4 Plasma pool pull‐down assay

A 3‐mL hypercholesterolemia plasma pool was generated as described above. To conduct the pull‐down assay, 300 µL of this plasma pool was incubated for 30 min at room temperature with either 1 µg anti‐human chemerin labeled with streptavidin (R&D Systems, Abingdon, UK) or 5 µg recombinant human chemerin‐His protein (Thermo Fisher Scientific). Subsequently, 50 µL of prewashed Dynabeads of streptavidin (Thermo Fisher Scientific) was added to the plasma pool with anti‐human chemerin labeled with streptavidin, and 50 µL of prewashed Dynabeads of His‐tag (Thermo Fisher Scientific) was added to the plasma pool with human chemerin‐His protein. The same amount of Dynabeads was also added to blank plasma. Plasmas were incubated overnight at 4°C while being rotated. Next, the Dynabeads were removed with a magnet, and washed four times with wash buffer (50 mmol/L sodium phosphate, pH 8.0 containing 300 mmol/L NaCl and 0.01% Tween‐20). Next, 50 µL elution buffer (identical to the wash buffer and additionally containing 300 mmol/L imidazole) was added to elute the pulled‐down proteins, which were collected for further western blotting. Western blotting was performed in parallel in the wash buffer and plasma remaining after the Dynabeads removal.

### 4.5 Biochemical measurements

TG, TC, LDL‐C, and HDL‐C were determined using standard clinical chemistry techniques at the Clinical Chemistry Department of the Erasmus MC. Chemerin in plasma samples, lipoprotein subfractions, and cell culture medium was assessed by a commercial enzyme‐linked immunosorbent assay (ELISA) according to the manufacturer's guidelines (R&D Systems nr. DY2324). The detection limit was 31.2 pg/mL. Plasma hsCRP levels were also quantified by commercial kit, according to the manufacturer's instructions (R&D Systems nr. DY1707). The detection limit was 15.6 pg/mL.

### 4.6 Western blotting and real‐time PCR

Protein levels from cell lysates were quantified using the BCA assay kit (Thermo Fisher Scientific). For immunoblotting analysis, cell lysates containing an equal amount of protein (10–25 µg), 10‐µL plasma pool samples, and 15 µL of wash buffer and pulled‐down samples were resolved through SDS‐PAGE and subsequently probed using primary antibodies as detailed in Table S3. HRP‐conjugated goat anti‐mouse or goat anti‐rabbit antibodies (Bio‐Rad Laboratories, Hercules, CA, USA) were then added, followed by detection with ECL (Bio‐Rad Laboratories).

Total RNA from the cell lysates was extracted using a commercial kit according to the manufacturer's instructions (Zymo Research, Orange, CA, USA), and reverse transcribed using PrimeScript RT Master Mix for real‐time PCR (Takara Shuzo, Kyoto, Japan). Real‐time PCR was performed using SYBR®Premix Ex TaqTM II kit (Qiagen, Venlo, The Netherlands) according to the manufacturer's instructions as described before.^43^ Primer sequences are listed in Table S4. Expression levels were determined using a standard curve, and human 36B4 was used as an internal control to normalize gene expression levels.

### 4.7 Statistical analysis

Statistical analysis was conducted using GraphPad Prism 9.5.0 (GraphPad Software, Inc., San Diego, CA, USA). Data of plasma are presented as mean ± standard deviation for normal distributions and median with range for non‐normal distributions. Data of cell experiments are presented as mean ± SEM. The normality of the data distribution was evaluated using the Kolmogorov‐Smirnov test. For the clinical data, variables were compared using either a paired *t*‐test or Wilcoxon test. Categorical variables were compared using the *χ* ^2^‐test. To examine the association between chemerin and other variables, Spearman's correlation analysis was performed, after correction for sex, age, and BMI. The levels of chemerin in lipoprotein subfractions was determined using two‐way ANOVA and Sidak's multiple comparison test. One‐way ANOVA was used to compare parametric data, while Kruskal–Wallis was used to compare nonparametric data for western blotting of cell lysates, qPCR data of HepG2 cells, ELISA's of the medium, and the percent of cholesterol efflux. *p* < 0.05 was considered statistically significant.

## AUTHOR CONTRIBUTIONS

Lunbo Tan, A. H. Jan Danser, and Koen Verdonk conceived the study. Xifeng Lu, A. H. Jan Danser, and Koen Verdonk supervised the study. Lunbo Tan, Na Wang, Leonie van Vark‐van der Zee, Monique T. Mulder, and Koen Verdonk designed methodology. Lunbo Tan, Na Wang, Annet M. H. Galema‐Boers, and Leonie van Vark‐van der Zee performed experiments, data collection, and data analysis. Lunbo Tan and Na Wang performed bioinformatics and statistical analyses. Annet M. H. Galema‐Boers and Jeanine Roeters van Lennep recruited patients, and collected clinical data. Lunbo Tan, A. H. Jan Danser, and Koen Verdonk wrote the original draft. All authors have read and approved the final manuscript.

## CONFLICT OF INTEREST STATEMENT

The authors declare no conflict of interest.

## ETHICS STATEMENT

The research described in this study was approved by the Medical Ethical Review Committee of the Erasmus MC, The Netherlands. The study was not subject to the Medical Research Involving Human Subjects Act, and received a waiver (MEC‐2016‐220). All patients gave informed consent. The study was conducted according to the Declaration of Helsinki.

## Supporting information

Supporting Information

## Data Availability

Data supporting the findings of this study will be made available by the corresponding author upon reasonable request.
