# Supplementary material for: Statins, but not proprotein convertase subtilisin‐kexin type 9 inhibitors, lower chemerin in hypercholesterolemia via low‐density lipoprotein receptor upregulation
Source: MedComm (2020). 2024 Aug 31;5(9):e681. doi: 10.1002/mco2.681 (PMC11364859; doi:10.1002/mco2.681)
Supplement: Supplementary file 1 — Supporting Information [file MCO2-5-e681-s001.docx]

**Supplemental Material for**

**Statins, but not proprotein convertase subtilisin-kexin type 9 inhibitors, lower chemerin in hypercholesterolemia via low-density lipoprotein receptor upregulation**

Running title: Statins, but not PCSK9i, lower chemerin

Lunbo Tan^1,2^, Na Wang^1,2^, Annet M.H. Galema-Boers^1^, Leonie van Vark-van der Zee^1^, Jeanine Roeters van Lennep^1^, Monique T. Mulder^1^, Xifeng Lu^2^, A.H. Jan Danser^1^, Koen Verdonk^1*^

^1^Division of Vascular Medicine and Pharmacology, Department of Internal Medicine, Erasmus MC, Rotterdam, The Netherlands.

^2^Clinical Research Center, The First Affiliated Hospital of Shantou University Medical College, Shantou 515041, China.

^*^Correspondence to: Koen Verdonk, MD, PhD. Email: k.verdonk@erasmusmc.nl.

**Table S1.** Characteristics of the hypercholesterolemic patients treated with both a statin and PCSK9i.

| **Factors** | **Number** | | | **Correlation with chemerin**  **(no adjustment)** | |
| --- | --- | --- | --- | --- | --- |
| Total | 23 | | |  | |
| Female (%) | 12 (52%) | | |  | |
| Age (years) | 61 (51-68) | | | 0.03 | |
| BMI (kg/m^2^) | 26.9 (24.7-30.2) | | | 0.10 | |
| Smoking ever (%) | 10 (43%) | | |  | |
| Mutation of LDLR (%) | 18 (78%) | | |  | |
| Myocardial infarction (%) | 7 (30%) | | |  | |
| Hypertension (%) | 11 (48%) | | |  | |
|  |  | |  | **Correlation with chemerin**  **(adjusted)** | |
|  | With statin | Follow-up with statin & PCSK9i | P-value | With statin | Follow-up |
| MAP (mmHg) | 99 (90-104) | 101 (92-107) | 0.44 | 0.04 | 0.04 |
| AST (U/L) | 25 (21-32) | 26 (20-32) | 0.78 | 0.04 | 0.13 |
| Triglyceride (mmol/L) | 1.33 (1.01-1.61) | 1.30 (0.83-1.51) | <0.05 | 0.54** | 0.55** |
| Total cholesterol (mmol/L) | 5.9 (5.0-6.9) | 3.3 (2.6-4.1) | <0.0001 | 0.11 | 0.27 |
| HDL-C (mmol/L) | 1.28 (1.16-1.49) | 1.46 (1.21-1.78) | <0.01 | 0.35 | 0.45* |
| LDL-C (mmol/L) | 4.3 (3.42-4.96) | 1.43 (0.76-2.22) | <0.0001 | 0.07 | 0.13 |
| ApoB (g/L) | 1.27 (1.08-1.48) | 0.59 (0.39-0.76) | <0.0001 | 0.03 | 0.21 |
| hsCRP (ng/mL) | 445 (149-1250) | 393 (187-1252) | 0.16 | 0.25 | 0.22 |
| Lp(a) (nmol/L) | 25.0 (5-140) | NA | NA | 0.30 | NA |
| Platelets (10^9^/L) | 250 (204-295) | NA | NA | 0.44 | NA |
| Chemerin (ng/mL) | 129 (98-154) | 124 (100-143) | 0.44 |  |  |

Values are presented as either N number (and percentage of total) or as median and interquartile range (25th percentile - 75th percentile). BMI, body mass index; MAP, mean arterial pressure; AST, aspartate transaminase; HDL-C, high-density lipoprotein cholesterol; LDL-C, low-density lipoprotein cholesterol; ApoB, apolipoprotein B; hsCRP: high-sensitivity C-reactive protein; Lp(a), lipoprotein(a); NA, not available. Statistical comparison was by Mann-Whitney U-test. The correlation with chemerin was analyzed by the Spearman’s method and adjusted for sex, age and BMI. * P<0.05, **P<0.01.

**Table S2.** Changes in chemerin according to type of lipid-lowering treatment in patients receiving either a statin or a PCSK9i.

| Type of treatment | Number of patients (%) | Chemerin | | P-value |
| --- | --- | --- | --- | --- |
|  |  | Baseline | After |  |
| Fluvastatin | 4 (16) | 81 (75-123) | 70 (42-79) | 0.07 |
| Simvastatin | 7 (28) | 98 (82-136) | 74 (41-93) | <0.01 |
| Atorvastatin | 8 (32) | 156 (130-179) | 94 (73-130) | <0.01 |
| Rosuvastatin | 6 (24) | 85 (80-91) | 94 (78-108) | 0.44 |
| Alirocumab | 19 (49) | 85 (76-108) | 93 (72-105) | 0.65 |
| Evolocumab | 20 (51) | 92 (67-113) | 81 (62-110) | 0.49 |

Values are presented as either N number and percentage of total or as the median and interquartile range (25th percentile - 75th percentile). Statistical comparison was by Mann-Whitney U-test.

**Table S3.** List of the primary antibodies used in the study.

| **Antibody** | **Dilution** | **Vender** | **Catalog no.** |
| --- | --- | --- | --- |
| Anti-Chemerin antibody | 1:1000 | R&D SYSTEMS | MAB2324 |
| Anti-ApoA-I antibody | 1:1000 | Proteintech | 66206-1-Ig |
| Anti-His-Tag antibody | 1:1000 | Proteintech | 66005-1-Ig |
| Anti-LXRβ antibody | 1:1000 | Proteintech | 60345-1-Ig |
| Anti-LXRα antibody | 1:1000 | Proteintech | 14351-1-AP |
| Anti-ABCG1 antibody | 1:1000 | Proteintech | 13578-1-AP |
| Anti-ABCA1 antibody | 1:1000 | R&D SYSTEMS | MAB72071-SP |
| Anti-LDLR antibody | 1:1000 | Proteintech | 10785-1-AP |
| Anti-SREBP2 antibody | 1:1000 | Proteintech | 28212-1-AP |
| Anti-HMGCR antibody | 1:1000 | Proteintech | 13533-1-AP |
| Anti-CMKLR1 antibody | 1:1000 | R&D SYSTEMS | MAB362-SP |
| Anti-CCRL2 antibody | 1:1000 | Proteintech | 66611-1-Ig |
| Anti-β-actin antibody | 1:5000 | Proteintech | 66009-1-Ig |
| Anti-GAPDH antibody | 1:5000 | Proteintech | 60004-1-Ig |
| Anti-GRP78 antibody | 1:1000 | Proteintech | 11587-1-AP |
| Anti-CHOP antibody | 1:1000 | Proteintech | 15204-1-AP |
| Anti-ATF6 antibody | 1:1000 | Proteintech | 24169-1-AP |
| Anti-PERK antibody | 1:1000 | Proteintech | 20582-1-AP |
| Anti-p-EIF2S1 antibody | 1:1000 | Proteintech | 28740-1-AP |
| Anti-EIF2S1 antibody | 1:1000 | Proteintech | 11170-1-AP |
| Anti-XBP1S antibody | 1:1000 | Proteintech | 24868-1-AP |

**Table S4.** Primer sequences of qPCR.

| **Gene** | **Primer sequences (5’-3’)** |
| --- | --- |
| Human RARRES2 (Chemerin) | F: TGGAAGAAACCCGAGTGCAAA |
|  | R: AGAACTTGGGTCTCTATGGGG |
| Human LDLR | F: CTACAAGTGGGTCTGCGATG |
|  | R: TTTGCAGGTGACAGACAAGC |
| Human 36B4 | F: TCTACAACCCTGAAGTGCTTGAT |
|  | R: CAATCTGCAGACAGACACTGG |
| Human SREBP2 | F: AACGGTCATTCACCCAGGTC |
|  | R: GGCTGAAGAATAGGAGTTGCC |


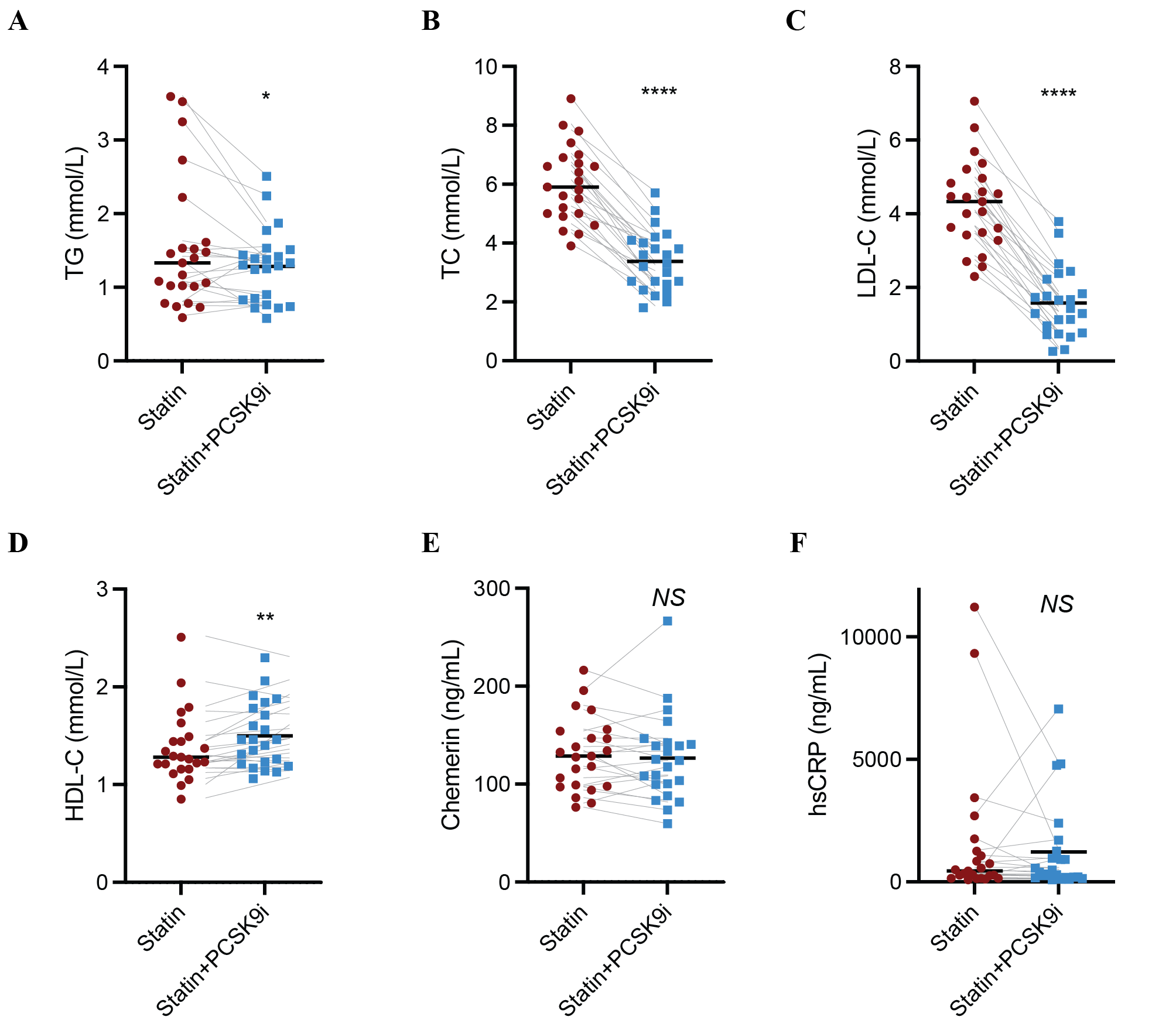


**Figure S1. Lipids and inflammatory markers in 23 hypercholesterolemic** **patients receiving a PCSK9i on top of a statin.** Panels A-F display the individual levels and median values of plasma triglycerides (TG), total cholesterol (TC), low-density lipoprotein cholesterol (LDL-C), high-density lipoprotein cholesterol (HDL-C), chemerin and high-sensitivity C-reactive protein (hsCRP). NS, not significant, *P<0.05, **P<0.01, and ****P<0.0001 versus with statin only treatment.

**
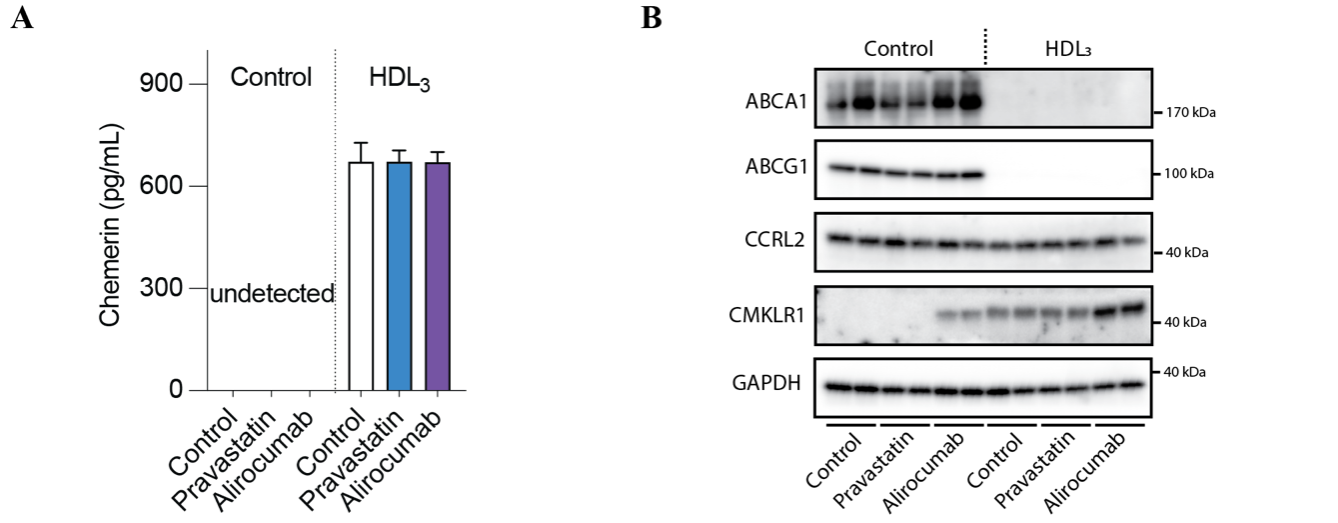
**

**Figure S2. The effect of HDL_3_ on cholesterol efflux and chemerin receptors in THP-1 cells.** THP-1 cells were cultured for 24 hours, in the presence of either 0.121 g/mL KBr (control) or 10 times diluted HDL_3_ isolated via ultracentrifugation from the plasma pool, with or without 3 μmol/L α-NETA, 20 μmol/L pravastatin, or 30 μg/mL alirocumab. Panel A, chemerin levels in the cultured medium; panel B, western blot images. Data are mean±SEM of n=3. KBr, potassium bromide; ABCA1; ATP Binding Cassette subfamily G member 1, ABCG1; low-density lipoprotein receptor, LDLR; chemerin chemokine-like receptor 1, CMKLR1; C-C motif chemokine receptor-like 2, CCRL2.


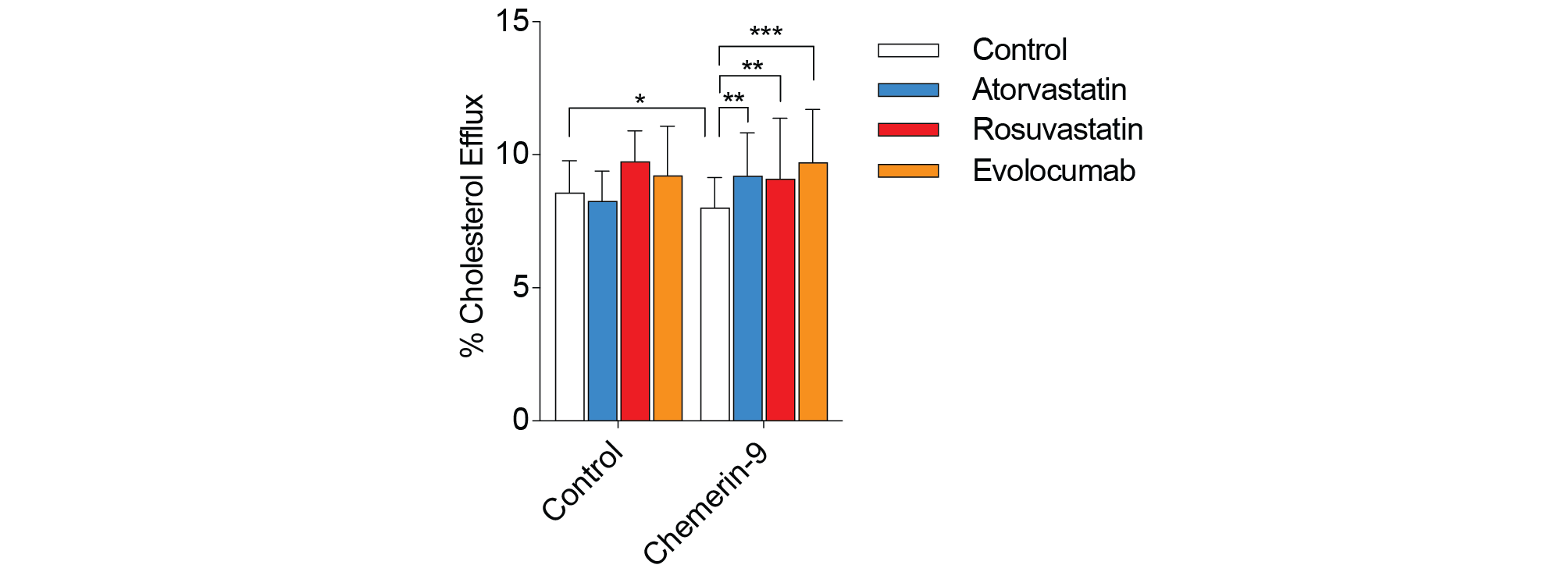


**Figure S3. The effect of atorvastatin, rosuvastatin and evolocumab on cholesterol efflux in differentiated THP-1 cells.** Chemerin-9 (100 nmol/L) was used to assess the effect of chemerin on cholesterol efflux from differentiated THP-1 cells, in the presence or absence of 100 μmol/L atorvastatin, 50 μmol/L rosuvastatin, or 50 μg/mL evolocumab, using 2 μL human serum as cholesterol acceptor. Efflux was calculated as medium fluorescence/(medium fluorescence + cell lysate fluorescence). Data are mean±SEM of n≥5. *P<0.05, **P<0.01, ***P<0.001 versus chemerin-9 alone.


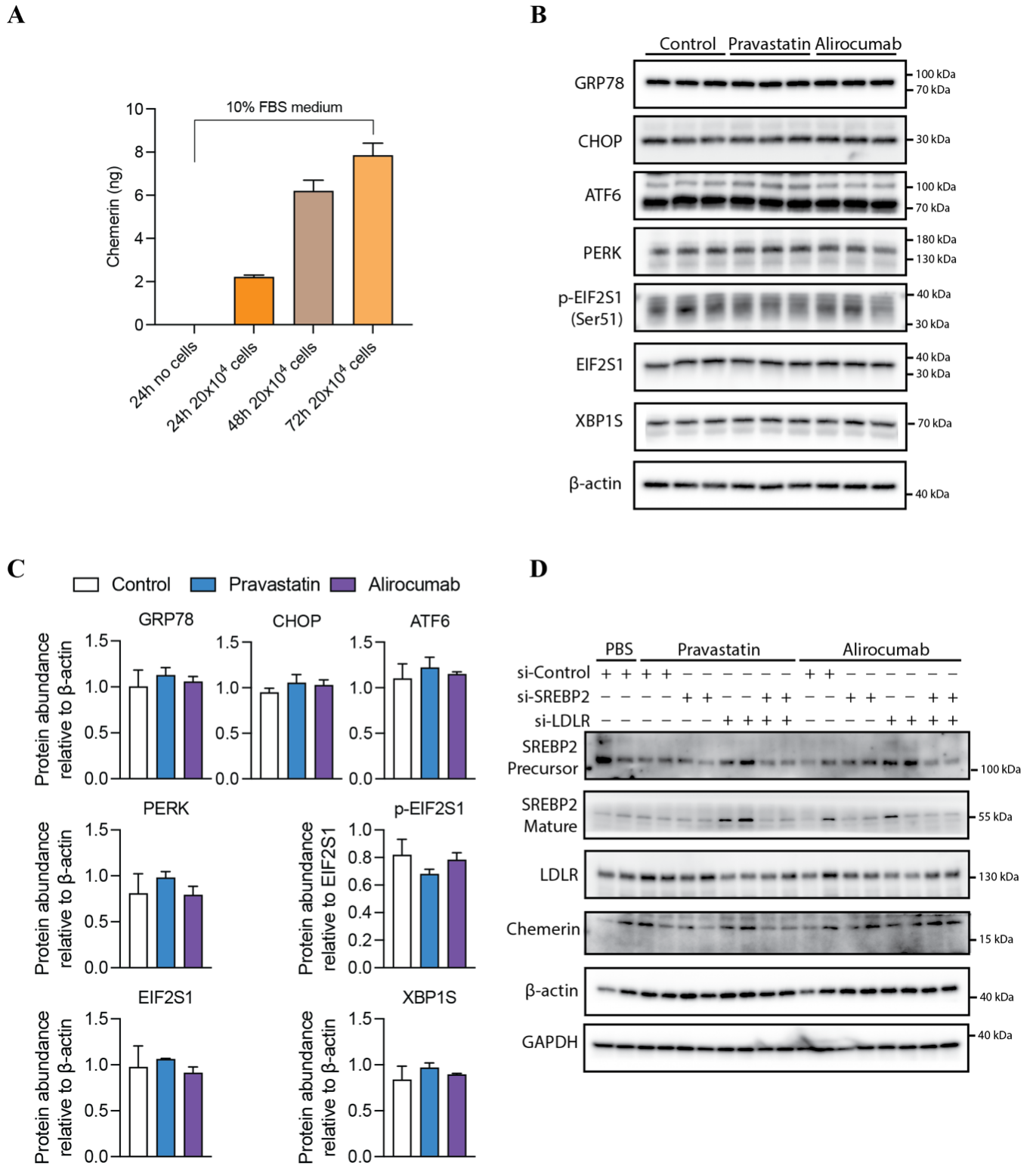


**Figure S4. The abundance of unfolded protein response-related proteins and SREBP2/LDLR RNA interference-related proteins in HepG2 cells.** HepG2 cells were cultured for 48 hours, in the absence (control) or presence of 50 µmol/L pravastatin or 30 µg/mL alirocumab. Panel A, chemerin content of the medium (0.5 mL) in the presence or absence of Hep2G cells, after 24, 48 or 72 hours of culturing without pravastatin or alirocumab. Data are mean±SEM of n=3. Panel B, western blot images, panel C western blot analysis. Data (mean±SEM, n=3) have been normalized to β-actin, except for p-EIF2S1, which was normalized to EIF2S1. Panel D, SREBP2 and LDLR knock-down in HepG2 cells under the following conditions: absence of treatment (control), treatment with 50 µmol/L pravastatin, or treatment with 30 µg/mL alirocumab for 48 hours. Glucose-regulated protein 78, GRP78; CCAAT-enhancer-binding protein homologous protein, CHOP; activating transcription factor 6, ATF6; protein kinase R-like endoplasmic reticulum kinase, PERK; eukaryotic translation initiation factor 2 subunit 1, EIF2S1; X-box binding protein 1, XBP1S; sterol-regulatory-element-binding protein-2, SREBP2; low-density lipoprotein receptor, LDLR.


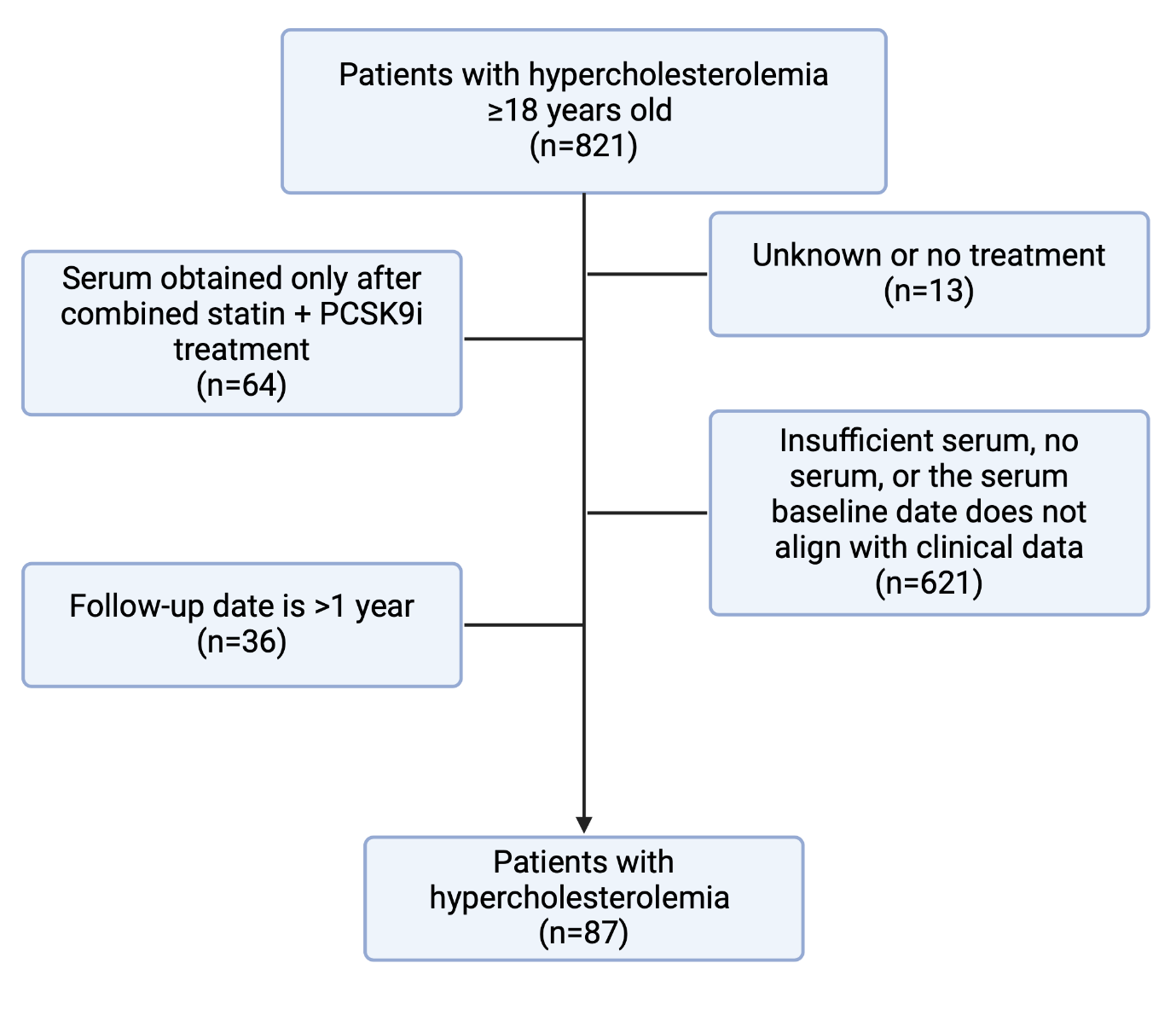


**Figure S5. Flowchart describing the patient inclusion.**
